# Atlanta Society of Medicine

**Published:** 1894-09

**Authors:** 


### *SOCIETY REPORTS.*

### ATLANTA SOCIETY OF MEDICINE.

ATLANTA, June 19, 1894.

*President J. W. Duncan in the Chair.*

Subject for the evening's discussion :

CONSERVATISM IN ABDOMINAL AND PELVIC SURGERY.

(See papers of Dr. Olmsted and Dr. McRae, pages 392 and 395.)

DR. JAS. B. BAIRD, in continung the discussion, said that he would express only his individual views without reference to the opinions or practices of others. Conservatism is the glory of modern medicine. The proudest claim of general surgery is that by improved and prudent methods it may now spare where it once condemned. I fear that operative gynecology does not deserve its full meed of praise for the exercise of that virtue which we call conservatism. Its achievements have been brilliant, we all admit, but has it not suffered itself to be blinded by enthusiasm and made reckless by ardent emulation and ill-advised rivalry? Opening the abdomen is now treated almost as a jest. From every city and town we read reports of consecutive laparotomies. Abdominal section is a grave and dangerous operation, and should not be undertaken except for good and sufficient reasons. I am among those who believe that the proper place for healthy ovaries is in the pelvis of the female, and not in the pocket of the gynecologist. The effects of castration in women are often prolonged and distressing vaso-motor disturbances, a tendency to morbid brooding, melancholy, suicidal impulses, and even insanity. An inevitable and deplorable result is sterility, often the cause of great sorrow. During the period of woman's menstrual life her mental, physical and social welfare depends largely upon the continuance of her catamenial and reproductive functions. Hence, the conservation of the organs which preside over these functions is of the utmost importance. (Dr.

Wm. Goodell on the "Conservative Treatment of the Female Pelvic Organs.") Yet, Mr. President, in my condemnation of an indiscreet and unwarranted resort to radical surgical measures, and in my plea for conservatism in the application of extreme procedures, I would not be misunderstood. Even extreme measures have their proper place, and in appropriate cases should not be rejected. It should be acknowledged that they are conservative, both in a technical and in a practical sense, when judiciously employed, and blind and unreasoning hostility to radical measures of treatment is reprehensible, as is an indiscriminate use of them. It is this routine, hasty, indiscriminate and unjustifiable use which constitute the cruel error of the present day.

Dr. Baird was present and was an active member of the old Atlanta Academy of Medicine when Dr. Robert Battey reported for the first time his first case of what he then called "Normal Ovariotomy." The discussion which followed was spirited.

It was then predicted that the operation would be greatly abused. In his opinion it is abused, as are some other operations involving abdominal section. He thinks there is too much operating—too great a tendency to follow the fashion—and it is his firm conviction that the fault may generally be laid at the doors of ambitious men who pursue special lines of work. He believes the world would be better off with less operating. These operations should be restricted; their application should be greatly modified. They should not be abandoned. The word conservatism has a definite signification. It means moderate, safe, prudent, opposed to that which is revolutionary, radical, extreme.

After all, the decision in individual cases must be left to the judgment of the operator and his associates. But the judgment should be educated and trained by correct and conservative habits of thought. Otherwise, even a conscientious man is liable to be led astray. He believes in proving all things and holding fast to that which is good.

DR. CROW said the most heroic measures are sometimes conservative. Operative interference is the only rational treatment in the presence of acute septic peritonitis from any cause, and especially from rupture of a pus tube, ovarian cyst, or in sepsis from a suppurating fibroid or myoma. In the majority of cases this is more successful than the expectant plan.

Another class of cases to which we apply this term, conservatism, includes diseased conditions of the uterus and appendages, with the adjacent structure and the neuroses. The treatment of this class has given rise to all kinds of fads, from electricity, massage, local applications to the uterus and vagina, to operative interference, from the removal of a cicatricial tissue in the cervix, curettement and repair of the perineum to opening the abdomen and removing a portion or the whole of the uterus and appendages. This is the class that I suppose we have reference to in the discussion for the evening, and it is a subject that has provoked the most animated discussions.

Dr. Polk, of New York, construes conservatism to mean leaving as much of the tubes and ovaries as we possibly can. Baldy, of Philadelphia, differs with him, and reports twelve cases of the partial removal of the appendages. Three of these were forced to have second operations, and the other nine remained unrelieved.

I am inclined somewhat to the views of Dr. Polk in a particular class of these cases; but in many cases I am frank to confess that nothing short of total extirpation will give the same amount of relief.

There is still another class that we often meet with, that I consider it the height of conservatism to let alone, so far as operative interference is concerned. These cases are largely *neurotic.* At times they suffer intense pain, the ovaries become extremely sensitive, but by studying these cases we are often led to believe that the pain is more the manifestation of nerve irritation, referable to conditions outside of the abdomen or pelvis, than to any immediate disease of the uterus or appendages, and by putting them under more favorable conditions we get a marked improvement in their supposed diseased condition of the ovaries.

I wish to refer to one thing in regard to minor pelvic surgery that is applicable to the general practitioner, that is, the necessity of immediate repair in all cases of extensive laceration of the cervix and perineum in childbirth.

I am convinced more and more each year that the neglect of this precaution is not only a lack of proper regard for conservatism, but is responsible for a large per cent. of our cases of puerperal sepsis, cellulitis, ovaritis and tubal trouble, which, after a time, necessitates the consideration of a radical operation as a means of relief.

DR. HARDON: Both sides have been presented well by men who operate and those who do not. Abdominal and pelvic surgery is a large field. It includes gynecology and the work of general surgery. In ovarian cysts and various tumors no rational man will object to their removal; the radicalism of to-day is the conservatism of to-morrow. There are tubes incurably diseased which should not be removed, yet there are those so diseased that it is folly not to remove them. Many cases of diseased tubes with repeated attacks of pelvic peritonitis require removal for relief.

Another class of diseased tubes arises from repeated simple inflammations. I am growing less inclined to remove tubes with chronic inflammation where there is no suppuration or infectious process present. Many cases of severe pain of ovaries cannot be relieved by any treatment, even in some by their removal, so I do not know where the line of conservatism lies. Some few will be relieved by operation—others will not; shall we give the patient the benefit of the doubt, or will we let them suffer and become habitual opiate users? Many cases of appendicitis will get well without operation, while some cannot without operation. In the case reported to-night by Dr. McRae it was impossible for the patient to recover without operation. The mortality of early operations is very low; later it is higher. It is better that one may die from operation occasionally than for many lives to be sacrificed for want of an operation.

In puerperal peritonitis many cases get well, but they are not cases of general peritonitis. A general peritonitis due to sepsis will die; it cannot be relieved by antipyretics, opiates, salts, etc. I have seen only two such cases get well, and they had the abdomen opened. When you see such cases growing worse, pulse rising and feeble, temperature rising, with indications of general peritonitis, operate. No medical treatment will reach or relieve these cases.

DR. KENDRICK: In regard to appendicitis I have had five cases in the last fifteen years with four recoveries, with only one case operated upon; one case, a woman with the third attack, was operated upon.

I agree with Dr. Hardon, where there is pus in the tubes they should be removed. In puerperal peritonitis the remedies that benefit such cases should be tried. In puerperal pelvic septic peritonitis had two cases; operated upon; found in one no pus, but found lymph, etc. Later, patient grew worse, and reopened; but found nothing in abdomen to indicate necessity for reopening.

DR. ELKIN: I regret that I did not hear that part of the papers. I believe in conservatism, yet we must not be too conservative. In ectopic pregnancy there is not much room for conservatism. Had an experience in my own family in which delay caused great risk. I think we have more mistakes made by waiting to see results than by early operation. Cases may die from the operation, but they are few. I think specialists are likely to be aggressive, but the general practitioner may be too conservative and wait too long for operative measures.

DR. HURT: I regret that I did not hear all of Dr. Olmsted's paper read. No field of surgery is more important just now than operations in the abdominal cavity. The transition from a limited knowledge and experience, with a greater per cent. of fatal results, to daily observations in this field and the use of antiseptics tending to a clearer elucidation of pathology and the removal of many fatal diseases, should not be abused by the over-ambitious, nor discountenanced by the timid and cautious. However, there is more danger in this day of operating without the necessity, than of waiting until a hopeless condition of pathology exists. Statisticians say that seventy-five per cent. of all cases of appendicitis recover.

Persistent treatment, both local and constitutional, has in my hands frequently relieved when I was about to despair of anything but the use of the knife. Septic peritonitis is apt to have succeeded the original location in the uterus or tubes, therefore the opening and washing out of abdomen does not reach all the septic matter, and it may not be sufficient to add to this the uterine douche. Many cases of so-called septic peritonitis find their origin in an entirely different pathology. I have known at least one uterus curetted, when in fact an inflamed mammary gland was the sole cause of fever. Barring cases of instrumental delivery, lacerations and adherent placenta, we seldom find cause for septicemia in the puerperal state. The uterus can in most cases, by the use of *vaginal antiseptic* washes, be made to expel clots of blood. I think we should not enter the uterine cavity with curette too hastily.

Dr. Kime: From the discussion it is evident that all agree that the operative measures should be resorted to when necessary. Then it is only a question as to when operation is necessary. That is a difficult question to decide, and cannot be definitely settled, as it depends upon the individual opinion of the operator.

In many pelvic troubles the indications for operative measures are plain, in others not so definite.

With infection after labor or abortion, it is imperative to clear and disinfect uterine cavity; if necessary use the curette. The physician that leaves the curette in his obstetric bag when its use is essential to save the life of the patient, is culpably negligent of his duty. Where, after dilating cervix, washing out uterus with disinfectant and curette forceps brings away decomposing débris, it should be removed with curette if it cannot be as efficiently done otherwise, thus preventing complications requiring celiotomy.

Where uterus is infected, walls breaking down, lymphatics, blood vessels or tubes and ovaries involved, it is useless to perform section without first correcting or removing the diseased and degenerated uterine structure. To remove the tubes and ovaries in such a case and leave behind other diseased structures would only invite disaster and failure. If the infection extends from the endometrium to the tubes and peritoneum, with uterine cavity cleared and disinfected, then section may be indicated. Also, where general peritonitis develops without serious involvement of other pelvic organs, celiotomy with flushing of abdomen is indicated.

To open abdomen in those cases where it is impossible to remove a degenerated uterus, infected tubes, with extensive adhesions and infection involving the lymphatic and blood vessels, is not to be counseled or advised. Even in extra-uterine pregnancy, operation is not *always* to be advised in *early* primary extra-peritoneal ruptures, and in cases where child has reached near the viable period, there are conditions which would favor delay in operative measures.

In appendicitis no one could consistently advise operative measures in all cases, while so many recover without it. Yet there are cases that demand operation which must be resorted to promptly if the patient is to be saved.

In closing the discussion Dr. Olmsted said: While confining my remarks to a limited phase of the subject, I quite agree with what has been said by Dr. McRae in regard to appendicitis, and also indorse some of the points made by Dr. Hardon in regard to certain conditions of the pelvic structures which require operative interference, but in regard to his cases of laparotomy for general puerperal peritonitis, would say that, from my standpoint, the two cases of recovery which he cites, I think recovered, not because of the operation, but in spite of it. The "*post hoc ergo propter hoc*" argument will not do. I think the two cases recovered by virtue of the "vis medicatrix nostrum," or, as religionists might put it, "By the grace of God." The gentleman (Dr. Hardon) has said that opium, quinine, etc., are not *remedies* in this condition, but I would like to remind him that while we have *no remedy*, strictly speaking, for typhoid fever, we yet, by the judicious selection of remedial agents and good general management, succeed in enabling our patients to weather the storm; so it is, I think, in general puerperal peritonitis, and I would remark that I have seen cases of this disease where septic conditions evidently existed, as shown by the fever, chills, etc., recover, and that too without the Epsom salts method of treatment, opium being the main stay. I regret that Dr. Hardon has not told us, in speaking of his cases of recovery, whether he has had any deaths after the operation, or how many he has known to die after its performance.

